# Two lenses, one disease: the power of joint working in SARD-ILD to ‘focus’ on the best care

**DOI:** 10.1093/rap/rkag058

**Published:** 2026-05-22

**Authors:** Laura White, Mia Rodziewicz, Lisa G Spencer, Elizabeth Perry

**Affiliations:** Lancaster University Medical School, Lancaster University, Lancaster, UK; Respiratory Department, Wythenshawe Hospital, Manchester, UK; Centre for Musculoskeletal Research, University of Manchester, Manchester, UK; Department of Respiratory Medicine, Aintree University Hospital, Liverpool, UK; Department of Rheumatology, University Hospitals of Bristol and Weston Foundation Trust, Bristol, UK

**This editorial refers to ‘Management of interstitial lung disease in systemic autoimmune rheumatic diseases: a survey of rheumatologists and pulmonologists’ by Genna Braverman *et al.*, 2026; https://doi.org/10.1093/rap/rkag050** 

Interstitial lung disease associated with systemic autoimmune rheumatic diseases (SARD-ILD) is estimated to account for 30% of all ILD [1]. SARD-ILD is a diverse group of conditions with variable pathogenesis, heterogeneous phenotypes and significant morbidity and mortality [2]. With advances in knowledge, disease awareness and the identification of novel autoantibodies associated with SARD-ILD, there is now more information to support the diagnosis, classification and management than ever before.

SARD-ILD guidelines were published by the ACR in 2023 [3] and the EULAR in 2024 [4]. These guidelines provide rheumatology and respiratory clinicians with evidence-based recommendations to support the approach to diagnosis, management and monitoring of SARD-ILD. Undisputedly, knowledge gaps still exist.

SARD-ILD sits at the intersection of rheumatology and respiratory medicine. With conditions that span two specialities, the risk of fragmented care is real.

## The current picture

In their survey of clinicians involved in SARD-ILD care, Braverman *et al.* [5] highlight how these specialities often approach the same disease through different lenses. Respiratory teams were more likely to favour corticosteroids and antifibrotic therapies, particularly when faced with progressive pulmonary fibrosis. Rheumatologists, by contrast, more commonly prioritised immunosuppressive or immunomodulatory treatment (IST). Such differences might be partly explained by the increase in number of ISTs available in recent decades and the paradigm shift towards ‘treatment to target’ and minimisation of corticosteroid exposure and toxicity.

The differing approaches were also reflected in the clinical factors that influenced treatment and management decisions, with specialists indicating greater ownership over facets of management most commonly performed within their speciality. Rather than representing disagreement, these findings underscore the complementary perspectives each specialty brings to the management of SARD-ILD. The results reinforce that effective care for these patients cannot be delivered within the confines of a single specialty.

Where Braverman *et al.* [5] highlight differences in the therapeutic approach, we must consider the ‘why?’ Although we have guidelines many recommendations are conditional with low or very low certainty of evidence [3, 4]. The heterogeneity of SARD-ILD and differences in pathophysiology are well recognised and yet studies frequently include multiple different SARD-ILDs and both inflammatory and fibrotic patterns of ILD. Perhaps it is unsurprising that the results are difficult to interpret with certainty and that in many subtypes of SARD-ILD we are reliant on extrapolation from studies in other SARD-ILDs.

## Monocular vision SARD-ILD care cannot remain in silos

ILD is one of the most serious and life-limiting manifestations of SARD. Yet, despite its complexity, care is still too often fragmented between rheumatology and respiratory medicine.

The survey by Braverman *et al.* [5] exposes this divide. These differences in approaches are not problematic; they are inevitable. What is problematic is the persistence of care models that expect either specialty to manage SARD-ILD in isolation. Such fragmentation is echoed by current patient experiences, whereby patients describe disjointed care, a lack of knowledge and a lack of involvement in care decisions [6]. Disjointed care risks delays in treatment, suboptimal management, disease progression and subsequently worse patient-related health outcomes.

## Refining the focus—the future of SARD-ILD care

SARD-ILD is not simply a rheumatological complication, nor merely a respiratory disorder. It is both. The implication is straightforward: patients require care that integrates expertise from both disciplines. When such models are implemented, the results are individualised, coordinated care for patients, without the burden of multiple specialist appointments [7].

The evidence base already supports multidisciplinary decision-making in ILD, where multidisciplinary discussion has become the diagnostic gold standard [8]. The same principle should extend more explicitly to management of SARD-ILD.

With the BSR expected to publish SARD-ILD guidelines this year [9], reflecting on the findings by Braverman *et al.* [5] and with the impact on patients from fragmented pathways, it is timely to consider how we support the best SARD-ILD care?

### How do we support the best SARD ILD care?

#### Making multidisciplinary assessment routine

Multidisciplinary care involving rheumatology and respiratory specialists should be routine for patients with SARD-ILD [9]. These conditions are complex and service design must facilitate closer working relationships. Clear referral pathways are essential in both directions: patients with autoimmune disease and respiratory symptoms should be assessed promptly by respiratory services and patients presenting with ILD should be evaluated for an underlying autoimmune rheumatic disease where needed.

Existing models for such collaboration include multidisciplinary team (MDT) meetings (co-located or virtual) and joint clinics [8]. MDT meetings will often include radiologists, rheumatology and respiratory physicians as a minimum and provide an opportunity for the evaluation of clinical cases through several lenses. Joint clinics between rheumatology and respiratory teams can also provide an efficient means of patient review and collaborative decision-making without the delays associated with e-mail or postal correspondence. In either case, explicitly coordinated shared pathways should align therapeutic decisions rather than leaving patients to navigate sequential, specialty-by-specialty care.

It is important to recognise that such services do not need to cost more money. Virtual MDT meetings can serve whole geographic regions. Respiratory and rheumatology consultants seeing a patient together in a joint clinic saves dual attendance, shares learning and supports unified management for patients, including the multiple facets of care integral to SARD-ILD. Rather than the purchase of a new house, it is more of a rearrangement of the furniture.

Equally important is a shift in professional culture and capability. As treatment options expand (targeted immunomodulators and antifibrotic agents), both specialties need to gain confidence working beyond traditional boundaries, with clear agreements on who initiates, monitors and escalates each therapy and how adverse effects are managed.

#### Building networks for all

Regional networks are essential to support local teams to deliver care close to home. They need to be inclusive. This enables access to specialist expertise for centres lacking locally established expertise and facilitates vital access to virtual MDT advice for complex or rapidly progressive disease.

Integrated models require practical enablers: protected time for MDT meetings in job plans, agreed referral/triage criteria and mechanisms to record and fund the multidisciplinary work that underpins safe, timely decisions. Knowledge must flow in both directions, empowering local teams to be able to support delivery of the best care, bringing care closer to home for patients in line with the National Health Service 10-year plan [10] and supporting dissemination and implementation of the latest evidence-based recommendations.

We must recognise SARD-ILD for what it is: a multisystem disease that cannot be optimally managed by one specialty alone. The goal should now be to make collaboration the default, with services designed to keep care coordinated, evidence-based and patient-centred.

#### Research agenda

Forming effective networks would not only deliver better care from the current evidence base but has the potential to shift the dial in terms of the evidence from which we make our treatment decisions.

To date many studies in SARD-ILD include heterogeneous groups, often with different SARDs, inflammatory and fibrotic pattern disease. Networks bring opportunities to develop registries, core datasets and translational research and to support collaborative studies (e.g. phenotype stratified trials and comparative effectiveness of clinical strategies). This research is essential to develop the evidence base to further improve patient outcomes with all subtypes of SARD-ILD and to move away from extrapolation and uncertainty.

The question is no longer whether collaboration improves care, it is why we have been slow to make it routine. We need to bring our teams together with a laser focus on improving SARD-ILD care.

## Data Availability

No new data were generated or analysed in support of this article.
